# Editorial for “Mechanical Behavior of Concrete Materials and Structures: Experimental Evidence and Analytical Models”

**DOI:** 10.3390/ma15144921

**Published:** 2022-07-15

**Authors:** Dario De Domenico, Luís F. A. Bernardo

**Affiliations:** 1 Department of Engineering, University of Messina, 98166 Messina, Italy; 2 Centre of Materials and Building Technologies (C-MADE), Department of Civil Engineering and Architecture, University of Beira Interior, 6201-001 Covilhã, Portugal; lfb@ubi.pt

Concrete is one of the most widespread materials in the civil engineering field due to its versatility for both structural and non-structural applications depending on the density range, competitiveness in terms of durability and manufacturing costs, as well as ease in finding raw constituent elements. For this reason, the mechanical behavior of concrete and, even more, reinforced concrete (RC) has been a research theme tackled by many researchers through different approaches for years. Although the relevant literature is full of papers on this topic, ranging from experimental works to theoretical contributions, an accurate and comprehensive description of the actual mechanical behavior exhibited by concrete and reinforced concrete at service and ultimate conditions still remains a challenge in the field of structural engineering. This is due to several intricate and interconnected phenomena involved, such as tensile cracking, compression crushing, strain softening, interaction between aggregates and matrix, interaction between concrete and reinforcement, stiffness degradation, energy dissipation, and ductility exhibited under cycling loading.

Following these research motivations, this Special Issue collects 15 papers focused on the mechanical behavior of concrete materials and structures, including both experimental findings and numerical analyses using both conventional and advanced methodologies. In the Editors’ opinion, each article contains clear scientific novelty from various standpoints (analytical, numerical, experimental, conceptual), thus representing a major contribution to the understanding of the mechanical behavior of concrete materials and structures. The Editors hope that this article collection can somehow contribute, even if modestly, to the continuous research for a more thorough and reliable understanding of the mechanical behavior of ordinary and prestressed concretes, as well as special concretes, including high-strength, recycled, and fiber-reinforced concretes, for both structural and non-structural applications, and for the development of related numerical/analytical predictive models.

Among the experimental contributions, Ni et al. [1] presented static and dynamic shear tests on four types of transverse connections used in adjacent precast concrete box-beam bridges to evaluate their shear transfer performance before and after cracking. In addition to experimental tests, a finite element model was also developed to calibrate and validate the interfacial material parameters. This contribution provided quantitative information on the effects of shear key cracking on vertical stiffness, and on the relationship between shear transfer and relative displacement across the shear key.

Three contributions were focused on recycled concretes, from either an experimental or numerical perspective. Garbaya et al. [2] incorporated phosphogypsum, which is a by-product of the production of phosphoric acid, into a new construction material. Based on an experimental campaign comprising physicochemical, mechanical, and thermal analyses, it was demonstrated that the different degrees of hydration that this material possesses facilitate the exchange of water with the external environment by creating a water pump that helps to condition the ambient air. Jahandari et al. [3] studied, through experimental tests, the compression behavior of concretes prepared with recycled coarse aggregates at replacement levels of 50% and 100% of natural coarse aggregates, resulting from both low- and high-strength concretes. The experimental campaign, including 60 tests, was aimed to analyze the effect of hooked-end steel fibers and silica fume, introduced as a partial replacement of cement, in such recycled concrete specimens. It was shown that the addition of steel fibers and silica fume considerably increased the strength (especially for recycled aggregates resulting from high-strength concretes), the elastic modulus, and the post-peak ductility of concretes. The third paper pertinent to the field of recycled concrete was authored by Peng et al. [4], who proposed a numerical analysis method for recycled concrete called the parallel homogenization method. An equivalent meso-damage model was developed through the base force element method, based on the complementary energy principle. The recycled concrete was treated as a five-phase medium, including the aggregate, the old mortar, the new mortar, the old interface, and the new interface. Based on the simulation of experimental uniaxial tensile tests of recycled concrete, it was demonstrated that the proposed method shows significant computational advantages over alternative mesoscopic damage models.

The mechanical behavior of concrete was analyzed not only under static loading. The dynamic characteristics of concrete were experimentally investigated by Sun et al. [5] through large-diameter split Hopkinson pressure bar (SHPB) tests performed on concretes and mortars comparatively. The experimental tests made it possible to analyze the influence of strain rate on the actual dynamic strength of concrete materials and the influence of strain acceleration on inertial effects. It was also shown that the strain rate effect of concrete is more sensitive than that of mortar, but the inertia effect of mortar is more sensitive than that of concrete.

Another aspect playing a key role in structural serviceability analyses of RC structures is the concrete shrinkage. Dey et al. [6] presented a comprehensive experimental program aimed to investigate the long-term (estimated at five years from casting) shrinkage effects of concrete on the deformative and tension stiffening response of RC members. Experimental tests on 14 RC ties with various geometry and mechanical characteristics demonstrated that the long-term shrinkage of concrete remarkably lowered the cracking load of the RC members and caused an apparent tension stiffening reduction, especially for members with higher reinforcement ratios.

The influence of multi-walled and single-walled carbon nanotubes (CNTs) on the carbonation, compressive and flexural strength, electrical resistance, and porosity of special mortars was experimentally analyzed by Lee et al. [7]. Based on the experimental outcomes, the introduction of CNTs led to a decrease in compressive and flexural strengths compared to plain mortars, due to an increase in the internal pore volume. However, the mortars prepared with CNTs exhibited a much lower electrical resistance (of around 10–20% of the plain specimens) and a higher acceleration rate of conductive cement mortar (the carbonation rate of conductive cement mortar increased by 1.5 times as the dosage of CNT was doubled in the mixture).

An increasing number of researchers have recently advanced the use of machine learning methodologies for predicting the mechanical characteristics of concrete based on training sets of data. For estimating the compressive strength of ultra-high-strength concrete (UHSC), Shen et al. [8] used soft computing techniques by considering 372 different mix proportions with 10 input variables, namely, cement content, fly ash, silica fume and silicate content, sand and water content, superplasticizer content, steel fiber, steel fiber aspect ratio, and curing time. The effect of these ten input parameters on the output parameter (compressive strength) and their interaction was evaluated using SHapley Additive exPlanations. It was demonstrated that the curing time has the highest impact on UHSC compressive strength estimation, followed by silica fume, sand, and superplasticizer content. Along a similar research line, Ahmad et al. [9] used supervised machine learning techniques, in particular, for comparing individual and ensemble algorithms to predict the compressive strength of fly-ash-based concretes, trained by 270 experimental data collected from the literature. In this study, the input parameters included cement content, aggregates, water, binder-to-water ratio, fly ash, and superplasticizer.

Bernardo et al. [10] addressed the problem of predicting the torsional strength of RC members through an analytical approach, inspired by the space truss analogy, with some empirical coefficients obtained by regression analysis. A wide database containing 202 tests of RC beams tested under pure torsion was first compiled, including under- and over-reinforced beams, plain and hollow beams, as well as normal- and high-strength concrete beams. Based on this database, correlation studies between the torsional strength and geometrical and mechanical parameters of the RC beams (compressive concrete strength, concrete area enclosed within the outer perimeter of the cross section, and amount of reinforcement) were carried out, following which refined predicting equations were elaborated to predict the torsional strength of RC beams. It was also demonstrated that the accuracy of the proposed equations is superior to that of alternative code-based formulations. The torsional behavior of RC beams, with particular attention to the transition from the uncracked to the cracked stage, was also investigated by Teixeira and Bernardo [11] through the generalized softened variable angle truss-model (GSVATM). The GSVATM was used to check the accuracy of some smeared constitutive laws for tensile concrete proposed in the literature. Among five different smeared constitutive laws analyzed in this paper, the formulation proposed by Belarbi and Hsu in 1994 exhibited the best accuracy and reliability against a wide database of experimental results including 103 RC beams with plain and hollow rectangular cross sections tested under pure torsion.

The deflection behavior of horizontal structural members was the object of the investigation of D’Antino and Pisani [12]. The recommendations of the various Eurocodes on the maximum deflection limits were critically analyzed by focusing on the integrity of the superstructures. Different types of horizontal members, namely, rib and clay pot (or hollow block), composite steel–concrete, and timber beam slabs, were designed to respect the deflection limit enforced by the Eurocodes. The authors proposed a curvature control method in place of the deflection control method adopted by the Eurocodes; this approach would allow for defining a general limit curvature value for floorings that could be adopted as the minimum performance level of the standards and would be able to guarantee the absence of flooring cracking.

One of the most complex phenomena to simulate in concrete structures is fracture behavior, due to the heterogeneous microstructure and interaction between aggregates and matrix. In this context, Tawfik et al. [13] employed state-of-the-art numerical techniques for simulating the fracture behavior of concrete. In particular, the authors proposed different crack simulation techniques, namely, the contour integral technique, the extended finite element method, and the virtual crack closure technique, implemented within the commercial finite element software ABAQUS, to investigate the flexural response and the fracture behavior of notched plain and reinforced concrete beams under three-point bending and four-point bending tests. The comparison of numerical outcomes with experimental findings demonstrated that the extended finite element method exhibited the best fracture energy estimation and solution-dependent crack path, and the most reliable results among the analyzed numerical techniques.

It is well known that durability of existing concrete structures was not always regarded as a crucial performance requirement in the past, and periodical maintenance plans have not been performed over the years. As a consequence, many damage phenomena have been recently observed in existing concrete structures dated from around 50 years ago; in some extreme cases, these damage mechanisms have led to complete structural collapse, as recently observed for bridges. One of the most serious factors negatively affecting the durability of concrete structures is the corrosion of steel reinforcement. In this context, De Domenico et al. [14] developed a systematic numerical-experimental approach for the load-bearing capacity assessment of existing prestressed concrete bridge decks in which the corrosion of prestressing strands was explicitly considered. The developed procedure can represent a convenient assessment tool to rapidly identify critical portions of a large infrastructure network prior to performing detailed analyses to establish a list of intervention priorities in a timely and reasonable way.

Finally, the review paper by Dong et al. [15] illustrated the various methods proposed in the literature to simulate the coupled hygro-thermo-mechanical behavior of concrete pavement. Among the analyzed methods, it emerged that an area of research that was not sufficiently explored by previous researchers is the deformation and failure mechanism of pavement concrete under the coupling action of moisture, temperature, and wheel load. In this context, COMSOL software was identified as a promising numerical tool for performing such coupled hygro-thermal-mechanical analyses of concrete pavement.
